# Genome-Wide Characterization of Heat-Shock Protein 70s from *Chenopodium quinoa* and Expression Analyses of *Cqhsp70*s in Response to Drought Stress

**DOI:** 10.3390/genes9020035

**Published:** 2018-01-23

**Authors:** Jianxia Liu, Runmei Wang, Wenying Liu, Hongli Zhang, Yaodong Guo, Riyu Wen

**Affiliations:** 1 College of Life Science, Datong University, Datong 037009, China; liujianxiashanda@163.com (J.L.); liujianxiashanda@sxdtdx.edu.cn (R.W.); lwylwy5@163.com (W.L.); bonus0301@163.com (H.Z.); 2 Maize Research Institute, Shanxi Academy of Agricultural Sciences, Xinzhou 034000, China; ymsgyd@163.com

**Keywords:** heat-shock proteins, *Chenopodium quinoa*, drought stress, phylogenetic analysis, synteny analysis

## Abstract

Heat-shock proteins (HSPs) are ubiquitous proteins with important roles in response to biotic and abiotic stress. The 70-kDa heat-shock genes (*Hsp70s*) encode a group of conserved chaperone proteins that play central roles in cellular networks of molecular chaperones and folding catalysts across all the studied organisms including bacteria, plants and animals. Several *Hsp70s* involved in drought tolerance have been well characterized in various plants, whereas no research on *Chenopodium quinoa* HSPs has been completed. Here, we analyzed the genome of *C. quinoa* and identified sixteen *Hsp70* members in quinoa genome. Phylogenetic analysis revealed the independent origination of those *Hsp70* members, with eight paralogous pairs comprising the *Hsp70* family in quinoa. While the gene structure and motif analysis showed high conservation of those paralogous pairs, the synteny analysis of those paralogous pairs provided evidence for expansion coming from the polyploidy event. With several subcellular localization signals detected in CqHSP70 protein paralogous pairs, some of the paralogous proteins lost the localization information, indicating the diversity of both subcellular localizations and potential functionalities of those HSP70s. Further gene expression analyses revealed by quantitative polymerase chain reaction (qPCR) analysis illustrated the significant variations of *Cqhsp70s* in response to drought stress. In conclusion, the sixteen *Cqhsp70*s undergo lineage-specific expansions and might play important and varied roles in response to drought stress.

## 1. Introduction

As sessile organisms, plants are constantly exposed to changing environments that impose stresses on their growth and development. Basic stresses such as drought, salinity can always cause cell injuries and produce secondary stresses to plants, both of which stimulate plants to synthesize a series of stress-responsive proteins to protect themselves [1]. Over millions of years, plants have evolved several strategies and morphological adaptations to tolerate these stresses. Heat-shock proteins (HSPs) are stress-related proteins that could be induced by almost all abiotic stresses. Since firstly discovered in *Drosophila* in 1960s, HSPs have been identified in all organisms [2,3,4]. As one of the stress-inducible proteins, HSPs are thought to be produced under non-lethal conditions to protect organisms from more severe stresses, termed as thermotolerance in plants, and have been proven to be the essential constituents in plants short-term tolerance to normally lethal temperature [4]. Discoveries in the past decades have shed some light on the importance of HSPs as molecular chaperones, which play diverse roles, even in unstressed cells, as molecular chaperones preventing the accumulations of other proteins and participate in protein refolding during heat stress conditions [5]. 

Based on their molecular weight, HSP superfamily proteins are grouped into HSP100, HSP90, HSP70, HSP60 and small heat-shock proteins (sHSPs). Among those groups, HSP70s have housekeeping functions in protein folding and protein quality control, resulting in preventing protein accumulations as aggregates and repairing misfolded conformers [6]. They assist a large group of protein folding processes in cells, and, during times of heat stress, certain *Hsp70s* are up-regulated and participate in the refolding of denatured proteins. The later identified HSP110 proteins (HSP110s) were also considered as members of the HSP70 superfamily based on the structural and functional similarities [7]. Structurally, HSP70s are comprised of an amino N-terminal adenosine triphosphatase (ATPase) domain and a carboxyl C-terminal peptide-binding domain [8,9]. Apart from the two conserved domain regions, the two extreme ends vary a lot in Arabidopsis as well as in other species, which were demonstrated to be related to the subcellular localization divergence of those proteins. In eukaryotic cells, members of HSP70 family localize to distinct subcellular compartments, including cytoplasm, plastids, mitochondria, and endoplasmic reticulum (ER) [10,11,12,13]. The various subcellular localizations are consistent with the functional divergences of HSPs, indicative of the complicated evolution history during evolution of eukaryotes [14]. The prokaryotic HSP70s are DnaK proteins, which are present under normal growth conditions and are induced by high temperature [4]. Several HSP70s have been well-characterized in a set of plant species. Bioinformatics analyses of the *Hsp70* gene family have identified 18 members in *Arabidopsis thaliana* [15], 24 copies of *Hsp70* in *Oryza sativa* [16] and 21 copies of *Hsp70* in *Physcomitrella patens* [17], while no research on *Chenopodium quinoa Hsp70* (*CqhsP70*) has been documented due to the lack of its complete reference genome. *C. quinoa* (quinoa hereafter) is a dicotyledonous pseudocereal and is an important crop with high nutrition [18]. Its gluten-free seeds contain an excellent balance of essential amino acids. Besides, elite traits, such as strong stress-tolerance, of quinoa make it one of the best species to elucidate the mechanisms underlying plant stress tolerance. The recently published high-quality reference genome of quinoa could substantially help to advance our understandings in stress resistance in quinoa [18].

To reveal the detailed evolutionary information of *Hsp70s* in quinoa, we identified sixteen Hsp70 members in newly sequenced quinoa genome based on well-known HSP70s in *Arabidopsis*. A more comprehensive phylogenetic tree of *Hsp70s* derived from 16 plant species was constructed, and the result indicated independent origination of those *Hsp70* genes before speciation of chlorophytes and the 16 *Cqhsp70*s were classified into eight paralogous pairs. Further analysis of those gene pairs showed high similarities in gene structure, in parallel to the conserved motifs in those paralogous pairs, indicating that the expansion of *Cqhsp70s* came from the recent polyploidy event. The synteny analysis of those scaffold containing *Cqhsp70s* suggested the chromosome-doubling event was the major force of *Hsp70* genes expansion after speciation of quinoa, termed as an allopolyploidy event. Furthermore, we analyzed the expression profiles of 13 *Cqhsp70s* in response to drought stress. Those expression analyses showed us various patterns of *Cqhsp70s* between and within *Hsp70* paralogous pairs, indicating the function diversity of those homologs in response to drought stress as well as other possible conditions during plants development. 

## 2. Materials and Methods

### 2.1. Identification of Heat-Shock Proteins 70 in Plant Species

To identify HSP70 members in the proteome of quinoa, protein sequences of 18 HSP70 members in *Arabidopsis* were obtained from TAIR10 (http://www.arabidopsis.org/). The 18 protein sequences were used as query to perform protein basic local alignment search tool (BLATP) search against the protein database of quinoa, with a maximum E-value of 1 × 10^−5^. Sequences were examined manually for apparent completeness and correctness against *Arabidopsis* HSP70 proteins in molecular evolutionary genetics analysis 7 (MEGA7) program [19]. To further verify the exact copy number of *Hsp70* gene, translated basic local alignment search tool nucleotide (tBLASTn) was also used to search other potential *Hsp70* genes in the genome of quinoa. To investigate the evolution history of HSP70 in plant species, HSP70 members in key plants were also identified based on AtHSP70 proteins. The following databases were used in these searches: *Chlamydomonas reinhardtii* [20], *Physcomitrella patens* [21], *Marchantia* *polymorpha* (http://marchantia.info/), *Selaginella moellendorffii* [22], *Amborella trichopoda* (http://amborella.huck.psu.edu/), *Spirodela polyrhiza* [23], *Zostera marina* (http://www.algaebase.org/search/), *Brachypodium distachyon* (http://www.plantgdb.org/BdGDB/), *Oryza sativa* (http://www.plantgdb.org/OsGDB/), *Glycine max* (http://www.plantgdb.org/GmGDB/), *Brassica rapa* (http://brassicadb.org/brad/), *Arabidopsis thaliana* (http://www.plantgdb.org/AtGDB/), *Arabidopsis lyrata* (https://genome.jgi.doe.gov/Araly1), *Chenopodium quinoa* from Phytozome v12 (http://phytozome.jgi.doe.gov/pz/), *Picea abies* (http://congenie.org/) and *Hordeum vulgare* (http://webblast.ipk-gatersleben.de/barley).

### 2.2. Alignments and Phylogenetic Analyses

For further analysis, all HSP70 protein sequences were subjected to multiple alignment using fast Fourier transform (MAFFT) [24] to generate multiple sequence alignments with default parameters (Supplementary Materials, File S1). ProtTest [25] was used to estimate which model of protein evolution fits the multiple sequences alignments. The phylogenetic tree was generated with PhyML [26], using the maximum likelihood method and best protein evolution model with 1000 bootstrap replications. The final tree was viewed and modified in MEGA7 [24].

### 2.3. Motif Search, Gene Structure and Synteny Analysis

Conserved motif searches were conducted on amino acid sequence alignments using the multiple EM for motif elicitation (MEME) tool from the MEME suite with default parameters [7]. Ten conserved motifs were detected in 16 HSP70 proteins from quinoa. The gene structure information of *Cqhsp70* family was obtained from annotation file of quinoa and was displayed using gene structure display server (GSDS) [27]. Predotar [28] was used to predict the subcellular localization of CqHSP70 proteins based on their protein sequences. To investigate HSP70 neighborhood microsynteny, the genome information of quinoa was used to detect the synteny and collinearity region for each *Hsp70* gene in their own scaffolds with multiple collinearity scan toolkit (MCScanX) [29]. Protein sequences from quinoa proteome were aligned against protein sequences in quinoa using BLASTp with a maximum E-value of 1 ×10^−20^. High-confidence collinear blocks with the score larger than 300 were selected according to the default parameters in MCScanX. The output image was constructed with Circos [30]. 

### 2.4. Plant Materials, Stress Treatment and Gene Expression Analyses

Quinoa plants were grown in the greenhouse. Seedlings were established under controlled conditions [31]: 60%–70% relative humidity, 14h of light and an average temperature of 23°C until two weeks, and then were irrigated with 25% (*w*/*v*) polyethylene glycol 6000 (PEG6000, Sigma-Aldrich, St. Louis, MO, USA) in soil homogenously. Above ground leave tissues mixed from five individuals at five-time points (0, 6, 12, 24, and 48 h) were collected during the treatments. Three replicates were performed from different batches of treatment. Total RNA was isolated from collected samples using TRIzol Up following the supplier’s instructions (Transgen, Beijing, China). RNA concentrations were measured using a Nanodrop-2000 spectrophotometer (Thermo Scientific, Wilmington, DE, USA). For each sample, 1 μg DNaseI-treated RNA were reverse-transcribed using the TransScript first-strand complementary DNA (cDNA) synthesis SuperMix (Transgen). Quantitative polymerase chain reaction (qPCR) was carried out in a 20 μL reaction mix using TransScript tip green qPCR SuperMix kit, following manufacturer’s instructions (Transgen). The Elongation Factor 1a (*EF1a*) was used as internal reference for normalization [32,33]. The gene primers used in this study are listed in Table S1. To compare the homolog gene expression patterns after drought treatment, we also took advantages of the available *Arabidopsis* transcriptomics data and analyzed *Athsp70s* expression profiles in response to drought treatment [21].

## 3. Results

### 3.1. Identification of HSP70 Proteins in Quinoa Proteome

**Figure 1:**
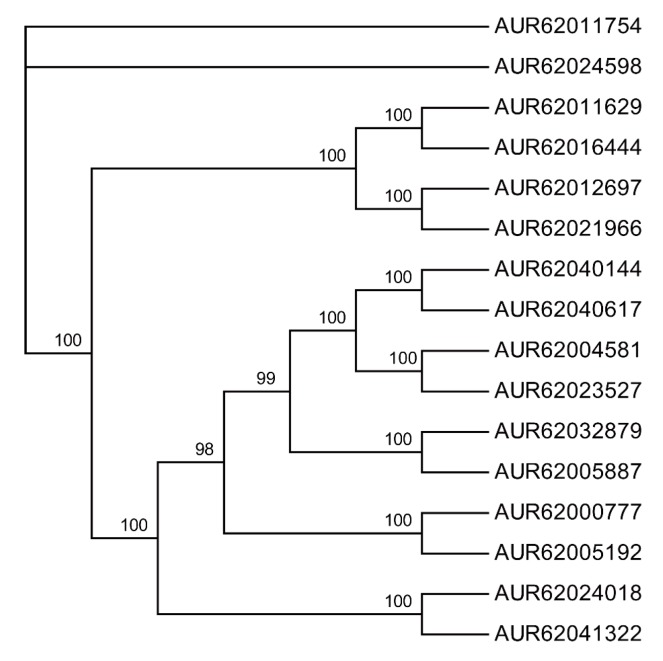
Phylogenetic tree of HSP70 in quinoa. Sixteen HSP70 homologs were identified in the proteome database of quinoa. Unrooted phylogenetic tree was constructed based on multiply-aligned sequences of the sixteen CqHSP70 protein homologs via PhyML using JTT substitution model with 1000 bootstrap replications as described in the method. Bootstrap values are indicated (in percentage) at the base of each node. With high bootstraps in each subclade, CqHSP70 members were classified as eight potential paralogous pairs.

To date, published plant genome-wide HSP70 superfamily information is restricted to only few species including *C. reinhardtii*, *P. patens*, *A. thaliana*, *O. sativa* and other model plants. Little is known about the HSP70 family in quinoa, one of the important and highly nutritious crops with tolerance to various stresses [22]. Eighteen AtHSP70s were used as query sequences in BLASTP searches against quinoa protein database. Only sixteen putative homologs were identified as HSP70 members in quinoa with a maximum E-value of 1 × 10^−5^. To date, the published reference genome of quinoa is of high quality with only 4.56% missing bases [22]. To verify the exact number of *Hsp70* genes in quinoa, BLASTn was used to identify other putative *Hsp70*-like genes. However, no other gene sequence with high confidence was obtained except for these 16 *Cqhsp70* genes. The HSP70s in quinoa ranged from 412 amino acids (aa) to 891 aa in length. To classify these 16 HSP70s, an unrooted maximum-likelihood tree was constructed based on the protein sequences of CqHSP70s (Figure 1), and the sixteen CqHSP70s were classified into eight paralogous pairs, which were further confirmed by the following analyses. 

### 3.2. The Land Plant HSP70 Superfamily Splits into at Least Seven Phylogenetic Clades

**Figure 2:**
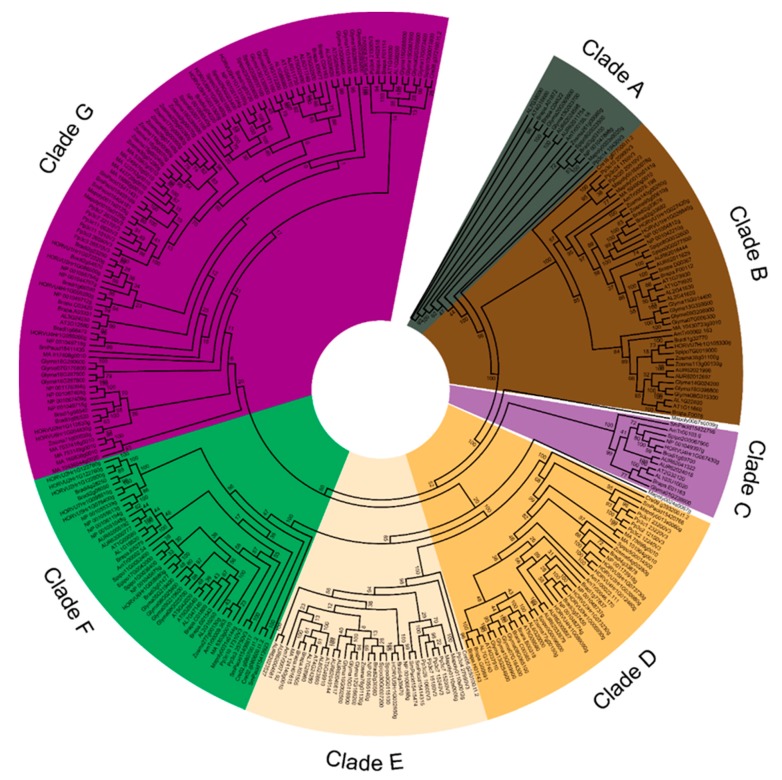
Phylogenetic tree of HSP70s in different plant species. Based on the total identified 293 HSP70 homologs in 16 plant species, an unrooted phylogenetic tree was calculated with the Maximum-Likelihood method, using JTT modeling with gamma-distributed rates and 1000 bootstrap replications. Bootstrap values are indicated at the base of each clade. The color region is associated with seven groups of proteins, i.e., Clades A to G.

Considering the broad dataset of sequences in different plant species and aiming to gain insights into evolutionary relationship of the HSP70 proteins, a more comprehensive phylogenetic tree of HSP70s was constructed based on protein homologs from a combination of 16 plant species, ranging from chlorophyte to seed plants. These species included: *C. reinhardtii* (Cre), *M. polymorpha (*Mapoly), *P. patens* (Pp), *S. moellendorffii* (Sm), *P. abies* (MA), *A. trichopoda* (AmTr), *S. polyrhiza* (Spipo), *Z. marina* (Zosma), *B. distachyum* (Bradi), *O. sativa* (NP), *H. vulgare* (HORVU), *G. max* (Glyma), *B. rapa* (Brapa), *A. thaliana* (AT), *A. lyrata* (AL) and *C. quinoa* (AUR). Based on our BLAST search, we identified 293 HSP70s in 16 analyzed species (Table 1). The numbers of HSP70 ranged from 6 in *C. reinhardtii* to 37 in *G. max*. Deviating from a former study, we detected 23, instead of 21 HSP70s, in the genome of moss *P. patens* [21]. All the newly identified HSP70s were subjected to detailed phylogenetic analysis. The full-length HSP70 proteins were aligned using MAFFT with default parameters and resulting alignments were used to generate unrooted maximum-likelihood phylogenetic tree with PhyML [34]. The final tree was comprised of seven clades (Figure 2 and File S2), termed Clades A–G. 

The HSP70s in green algae were found in Clades B, D, E, F and G, while Clades A and C only contain HSP70 proteins from land plants. Since *Hsp70* genes are universally present in all eukaryotes, the unrooted phylogenetic tree revealed the independent origination of those *Hsp70s* in chlorophyte, indicating an ancient origination and expansion events of *Hsp70* family prior the emergence of chlorophytes. Clade A contained 15 HSP70 members from 12 land plant species, which belonged to the *HSP110/SSE* subfamily according to previous report [19]. In this clade, no chlorophyte homolog was identified, whereas almost all land plants contained a single copy of *Hsp70* gene belonging to Clade A. Interestingly, there were two *Hsp70* genes in quinoa, soybean and rapeseed genomes in Clade A, which might come from independent duplication events after speciation. Clade B contained 45 HSP70 members with the emergence of *Hsp70* in chlorophyte. Clade C consisted of 12 members from lycophyte and seed plants. Clade D had 41 members from chlorophyte to seed plants. In this clade, only a single copy of *Hsp70* was retained in chlorophyte, liverwort and lycophyte, the other species contained two or more copies of *Hsp70*. The duplication event occurred prior to speciation of Brassicaceae while the other species contain multiple *Hsp70s* from independent duplication events. Clade E contained 33 members from chlorophyte to seed plants. Two or more copies of gene were retained in land plants after the independent duplication events, while one expansion event occurred before speciation of Poaceae. Clade F was comprised of 42 members. Clade G was the largest subfamily with 103 members in this group, whereas no *Hsp70* from quinoa was identified belonging this subfamily. 

Generally, whole genome duplication events were thought to occur two times. The first time happened during the evolution of the common ancestor of land plants, while the second event occurred during the evolution of the common ancestor of angiosperm plants [35,36,37]. These whole genome duplication events were the major force contributing to the expansion of genes-related to stress responses. In our phylogenetic analyses, we found that the second duplication event contributed to expansion of HSP70s in Clades B and F, while both genome duplication events were the reason for the expansion of HSP70 in Clade G. Apart from these two whole genome duplication events, species-specific duplication events played roles in the expansion of HSP70s in other clades, such as in Clades A, C and D. Many *Hsp70* paralogs in soybean were found to be classified into different clades in our phylogenetic tree due to whole genome duplication events, which was coincident to previous finding that the genome of soybean undergoes several times of genome duplications [38]. We also found that those 16 *Hsp70s* in quinoa formed eight paralogous pairs, indicating that recent duplication events or whole genome duplication events occurred after speciation of quinoa.

### 3.3. Gene Structure and Conserved Motif Analyses of Hsp70s and Their Encoding Proteins in Quinoa

**Figure 3:**
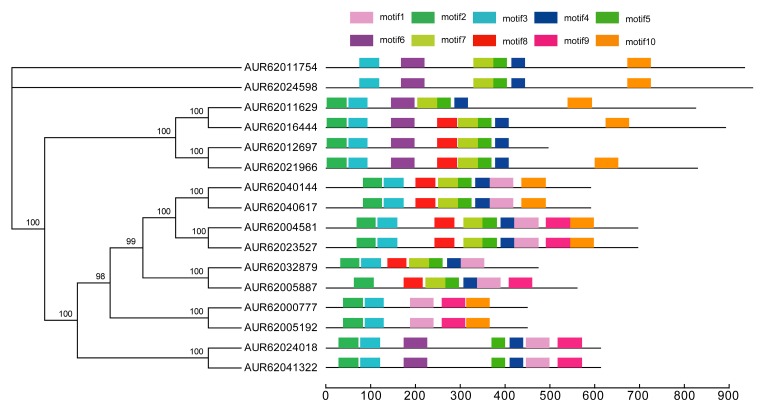
Conserved motifs across all CqHSP70s. Ten conserved motifs in all CqHSP70s were identified through the multiple EM for motif elicitation (MEME) analysis. Schematic representation shows conserved motifs and each motif is represented by a colored box numbered at the top. Scale bar indicates number of amino acids (aa).

To understand the functional diversification of CqHSPs during their evolution, 10 conserved motifs were detected in 16 CqHSP70s (Figure 3), in addition to the well-characterized N-terminal ATPase domain and C-terminal domain for protein-protein interaction. In our analysis, the recently duplicated CqHSP70 homologs, namely the eight paralogous pairs, exhibit similar motif arrangement architectures (Figure 3) in their protein structures. For all the 16 CqHSP70 proteins, motifs 2 and 3 exist in almost all of them, with only two exceptions: motif 2 is only absent in AUR62011754-AUR62024598 pairs and motif 3 is only absent in AUR6032879. The two conserved N-terminal motifs referred to the ATPase domain region are present in all the CqHSP70s, indicating the basically conserved biological functions of all HSP70s in quinoa. Motifs 4 and 5 are only absent from AUR62000777-AUR62005192 pair. No conserved motif region was detected in C-terminal region of all CqHSP70s, whereas this C-terminal region is conserved within the paralogous pairs. 

**Figure 4:**
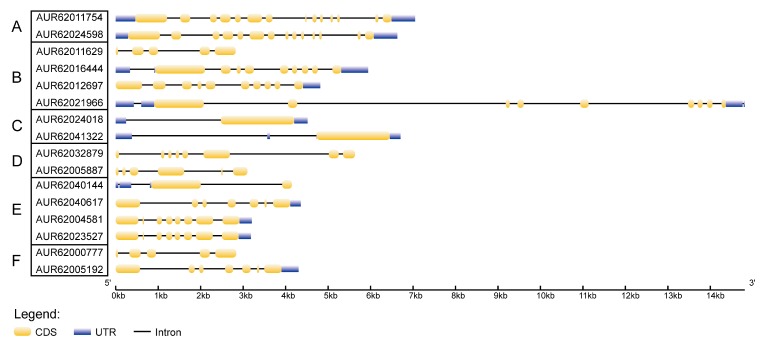
Conservation and diversity of gene structures of the *Hsp70* paralogous pairs in quinoa. This figure shows a schematic representation of gene structures of *Hsp70s* in quinoa. The yellow boxes represent exons, black lines represent introns and blue boxes represent untranslated region (UTR) region. The HSP70 Clades A–F (**A**–**F**) are indicated on the left. Scale bar indicates number of base pair (bp).

To better understand the evolution conservation of CqHSP70 family, we analyzed the gene structure of *Cqhsp70* genes based on available information from quinoa genome annotation file (Figure 4). The overall gene structures and the intron-exon numbers vary in these *Cqhsp70* genes, whereas the gene structure is highly conserved in the paralogous pairs from each clade derived from phylogenetic analysis. The gene structures vary significantly only in members from Clades B and D. This result suggested the disorder of the gene structure of *Hsp70s* in quinoa, indicating potential biological function diversities of those genes. 

To further investigate subcellular localization information of CqHSP70 proteins, Predotar prediction was used to predict the localization of these 16 HSP70s in quinoa based on putative target signals. AUR62011754, AUR62024598, AUR62000777 and AUR62005192 were predicted to localize in ER. AUR62040144, AUR62040617, AUR62004581 and AUR62023527 were assumed to localize to the plastid, with only AUR62032879 were predicted to localize to mitochondrial. According to previous report [19], the classifications of *Arabidopsis* HSP70 proteins in phylogenetic tree were corresponding to different subcellular localizations. Several signal peptides in *Arabidopsis* HSP70 proteins based on previous research [21,39,40] were present in our multiply-aligned protein sequences. For example, the C-terminal signal peptide his-asp-glu-leu (HDEL) was conserved in Clades B and F, indicating ER localization of these two groups of proteins. The C-terminal chloroplast-targeting signal (DVIDADFTD) was conserved in Clade E, with several position mutations occurring in two of the four CqHSP70 paralogous pairs. Members in Clade D were predicted to localize in mitochondrial based on two conserved signature sequences GDAWV and YSPSQI. The diversity of potential subcellular localization also provided us directions to a better understanding of the function diversity after duplication events of all the paralogous pairs.

### 3.4. Allopolyploidy Event Contributed to the Expansion of Cqhsp70s

**Figure 5:**
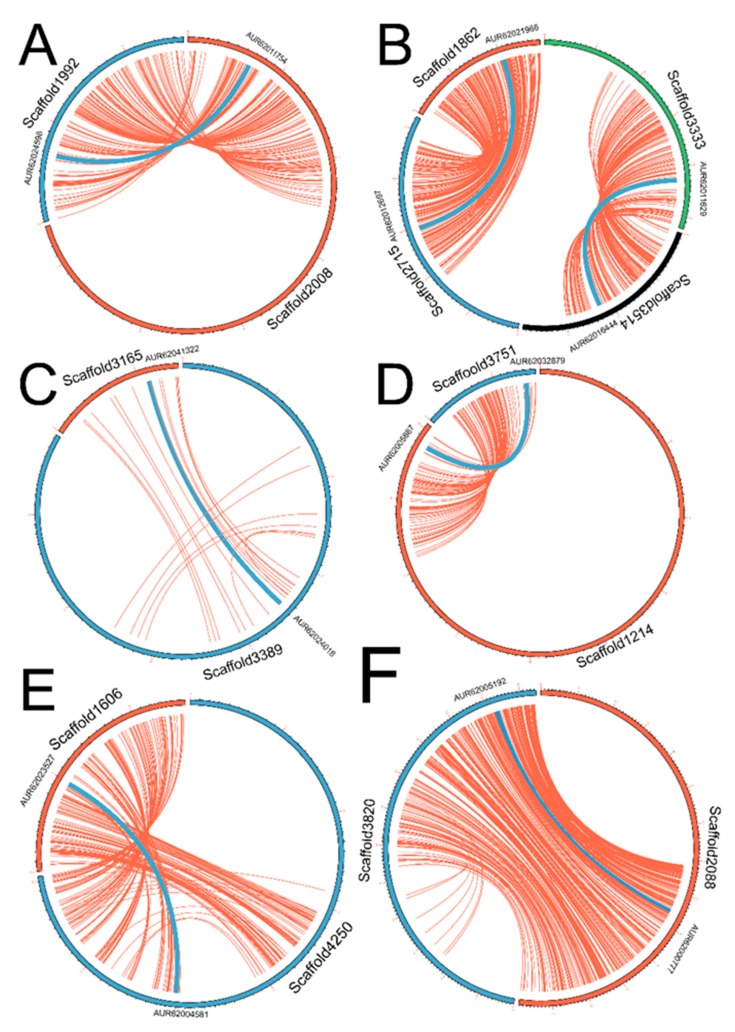
Synteny analysis of *Cqhsp*70. The synteny and collinearity regions of the *Cqhsp*70 paralogous pairs in their scaffold were performed with MCScanX [29]. Seven out of the eight pairs were used except the AUR62040144–AUR620440617 pairs whose scaffolds are too short to investigate the synteny region. The *Cqhsp*70 paralogous pairs were indicated by the blue lines while the red lines represented the conserved region in scaffolds. Note: (**A**)–(**F**) in this figure are consistent with the clade source of those paralogous pairs in Figure 2.

With independent origination of *Cqhsp70* genes before speciation of chlorophytes, another important question is to reveal the expansion history of *Hsp70* in genome of quinoa. The gene structure similarities and conservation of motifs between those paralogous pairs from the 16 *Cqhsp70s* suggested the potential duplication events in quinoa. Molecular phylogenetic analyses have revealed the allopolyploid origination of quinoa [22]. Considering the chromosome doubling events in allopolyploid and the later recombination and chromosomal rearrangement events occurred after the speciation of quinoa, we investigated the evolution history of CqHSP70 family. Our phylogenetic analysis has revealed the independent origination of those eight paralogous pairs in each clade (Figure 2). The gene structure similarities and protein motif conservations suggested HSP70s in quinoa expanded with recent duplication events. To reveal the detailed information, MCScanX was employed to investigate the homologs in those subgenome regions surrounding *Hsp70* in each scaffold. All sixteen *Cqhsp70* genes were mapped to each scaffold based on the publicly available information in the quinoa genome database, and the synteny analysis was performed with MCcanX. Synteny analysis revealed highly conserved regions surrounding those *Hsp70* pairs (Figure 5). Synteny information was obtained in seven out the eight pairs, except the small scaffold of AUR62040617-AUR62040144 pair. Relatively weak synteny regions were detected surrounding the pairs (AUR62024018 and AUR62041322) in Clade C, while high synteny regions were detected in other paralogous pairs. Considering the allopolyploidy event contributing to the speciation of quinoa, those paralogous pairs with high synteny region most likely came from chromosome-doubling event, and the chromosome-doubling event was the major force of expansion of HSP70s in quinoa. Apart from that, tandem duplications could be the reason for expansion of *Cqhsp70s* in Clade C, indicating that tandem duplications also played important roles in the gene expansion process.

### 3.5. Cqhsp70 Genes in Quinoa Are Responsive to Drought Stress

**Figure 6:**
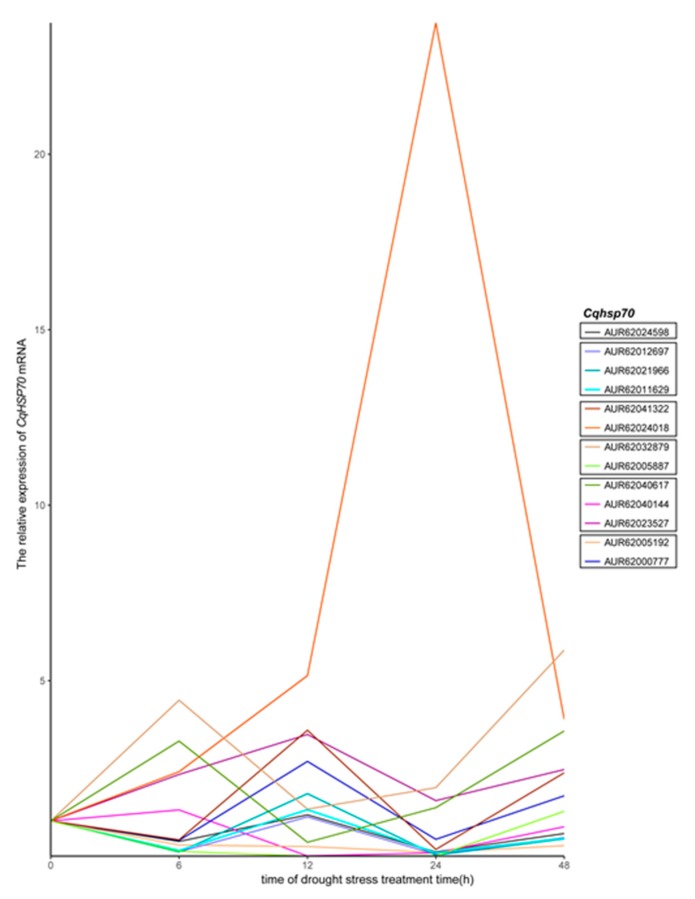
*Cqhsp70*s gene expression profiles in response to drought treatment. Two-week-old quinoa seedlings were irrigated with 25% PEG6000 (*w*/*v*) for drought stress. Above-ground tissues were collected at five time points (0, 6, 12, 24, and 48 h) during the treatments. Quantitative polymerase chain reaction (qPCR) assays were performed with different batches of treated plants and one representative data was shown.

Gene duplication event often leads to functional diversity [41]. To characterize the roles of *Cqhsp70s* in stress response, we isolated RNA from drought stresses-treated plants from different time points (Figure S1) and qPCR was employed to examine relative expressions of *Cqhsp70s* and then we could analyze the expression patterns of *Cqhsp70*s in above-ground tissues treated with drought stress (25% polyethylene glycol (PEG) *w*/*v*) (Figure 6). 

**Figure 7:**
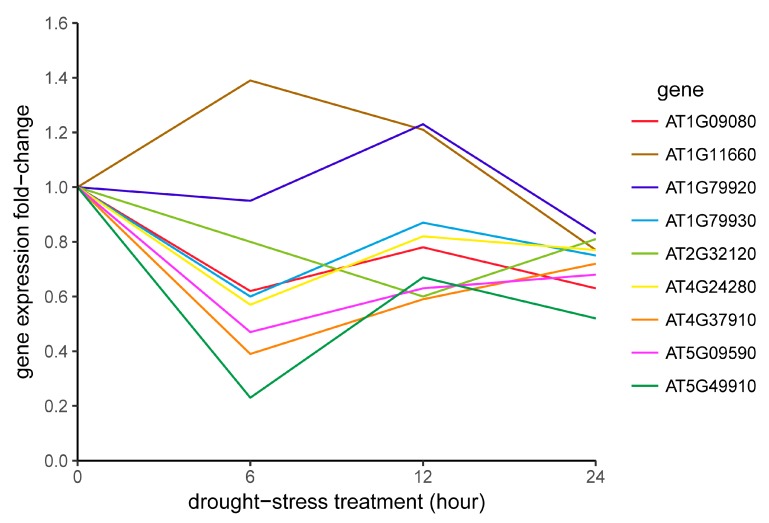
*Athsp70* gene expression profiles in response to drought treatment. The preliminary data were retrieved from the published transcriptomics data [21].

The expression profiles of their counterparts in *Arabidopsis* after drought stress were also retrieved for comparison (Figure 7) (Table 2) [21]. Expression of 6 out of the 14 *Cqhsp70*s was down-regulated at the beginning of drought stress treatment and recovered at 12h after treatment (Figure 6). In our data, about half of the 14 *Cqhsp70*s showed “drop-climb-drop” expression pattern, which was similar to the observations of their *Arabidopsis* homologs (Figure 7) (Table 2) [21]. While the expression of AUR62024018 maintained high expression as the time of treatments continued, AUR62041322 was up-regulated after 12h treatment. The expression pattern of two genes, AUR62032879 and AUR62005887, from Clade D varied from each other. The expression of AUR62005887 exhibited a drop after drought treatment and recovered at 48h after drought-stress treatment, whereas AUR62032879 was highly induced by drought and then dropped after 6h. AUR62040617 and AUR62040144, from Clade E, shared a similar expression pattern. The other two genes, AUR62023527 and AUR62004581, were highly induced by drought, while AUR62004581 was dramatically induced by drought treatment. For AUR62005192 and AUR62000777, in Clade F, both of the paralogs showed similar expression pattern with Arabidopsis orthologs and they were up-regulated 12h after treatment. These results suggested various roles of *Cqhsp70* gene sets in response to drought stress. 

Similar expression patterns of homologs from quinoa and *Arabidopsis* indicated the functional conservation of *Hsp70* genes, and we also found several special expression pattern of *Cqhsp70s* in drought stress conditions, suggesting diverged functions of HSP70s in quinoa.

## 4. Discussion

Since plants are continuously challenged by a variety of biotic and abiotic stresses, they have evolved stress tolerance strategies to compromise damages [42]. HSP70 is one of the widely conserved proteins that play important roles in plant stress tolerance. Gene replication are well-known to function importantly in the evolutionary process [41]. As one of the most ancient proteins, HSP70s are present in prokaryotes as well as eukaryotes. The ancient origination of HSP70s and the later expansion of this family give this family of proteins much potential ability of sub-functionalization and neo-functionalization. HSP70 is well-documented to be up-regulated in heat stress conditions in plants. Now, more and more reports revealed that HSP70 proteins play important roles in various stress response processes as well as are involved in plant developmental processes. However, the relationships of the early diverging eukaryote lineages, as well as the detailed origination and expansion history of HSP70 are still uncertain. Enabled with the well-characterized HSP70 family in Arabidopsis and the comprehensive genome information from different plant species, the evolution history of plant HSP70s would be clearer.

Quinoa is one of the elite crops with excellent stress tolerance ability under drought, high temperature and other adverse conditions. The stress tolerance in quinoa was generally considered to be associated with the expansion of genes involved in stress responses [43]. *Hsp70* genes are one of those important genes in stress responses. The information of recently sequenced quinoa genome allows us to unveil the detailed evolution information of *Hsp70* genes in this nutritious and stress resistant crop [22]. In this study, we identified sixteen *Hsp70* genes in the genome of quinoa based on the protein sequences of the 18 HSP70 in *Arabidops*is with BLASTp searches. To verify the exact numbers of *Hsp70* genes in the high-quality reference genome of quinoa, the nucleotide sequences of the 18 *Athsp70* genes and 16 *Cqhsp70* genes were also used to search against the whole genome of quinoa. However, no other fragment in the genome showed high similarity with the 16 *Cqhsp70* genes, suggesting only 16 *Cqhsp70* genes existing in the sequenced genome of quinoa until now.

Further phylogenetic analyses based on protein sequences of HSP70s in major plant species classified the total HSP70s into eight groups (Figure 1 and Figure 2). In our analysis, the *hsp70s* copy numbers vary from chlorophyte to higher plants such as Arabidopsis, ranging from 6 in chlorophyte to 37 in soybean. Phylogenetic analysis classified the six *Hsp70s* in chlorophyte into five clades, indicating that several copies of *Hsp70s* were present in the ancestor of chlorophytes. *Hsp70s* have been identified in other eukaryote species such as human and fruit fly, and in many prokaryotes including bacteria species. Those results proved an ancient origination of *Hsp70s* in living organisms and the independent originations of *Hsp70s* superfamily during the evolution of plant kingdom. While only six copies of *Hsp70s* were retained in the genome of chlorophyte, a larger amount of *Hsp70s* were identified in land plant species, especially in higher plants. In general, whole genome duplications and segmental duplications are the major force for the expansion of genes [35]. Previous research has proven several whole genome duplication events occurred during the evolution of plants, especially in the Cretaceous-Palaeogene (K-Pg) extinction time [44]. We also found several expansion events that contributed to the expansion of Cq*hsp70s* family, as shown in Figure 2. Further motif analyses and gene structure analyses showed high similarities with each of the eight paralogous pairs in quinoa, suggesting a recent genome duplication event for the expansion of *Hsp70s* in quinoa. 

Chromosome rearrangement and gene duplication events always follow the whole genome duplication events or polyploidy events. To verify our hypothesis, synteny analysis was performed based on the available gene location information from quinoa database. The synteny information was obtained for the seven out of the eight paralogous pairs. Only one pair from Clade C showed weak synteny relationship between the two scaffold regions surrounding the two paralogs, AUR62041322 and AUR62024018, and all the other pairs are located on the high-synteny scaffold regions, suggesting the potential polyploidy event or the later segmental rearrangement events were the major force of the expansion of *hsp70s* during the evolution of quinoa. Except for polyploidy event, tandem duplication could be one of the possible explanations for the expansion of *Hsp70s* in Clade C.

It has been demonstrated that *Hsp70* genes are involved in plant stress responses [21,45,46,47]. Since eight paralogous pairs of *Hsp70s* are present in the genome of stress resistant crop quinoa, we also investigated the potential roles those *Cqhsp70*s in stress responses as revealed by qPCR assay. The results demonstrated that the expression of some *Cqhsp70* genes were significantly altered in different stress stages. This was mostly consistent to previous transcriptomics data derived from model plant *Arabidopsis*. The different expression patterns of *Cqhsp70s* in response to drought stress indicated the various roles of these genes in drought stress tolerance, as well as the functional diversity of the multiple copies of *Hsp70* genes in quinoa, which could be one of the main reasons to explain the strong stress-tolerance ability of quinoa.

## Figures and Tables

**Table 1:** Heat-shock proteins 70 (HSP70) family members in selected plant species.

Plant Species	Number	Phylogenetic Class							Other
		I	II	III	IV	V	VI	VII	
*Chlamydomonas reinhardtii*	6	0	1	0	1	1	2	1	
*Metrosideros polymorpha*	11	1	2	0	1	2	1	2	2
*Physcomitrella patens*	23	1	3	0	4	5	2	8	
*Selaginella moellendorffii*	10	0	0	1	1	2	1	4	1
*Picea abies*	16	0	2	0	2	1	1	10	
*Amborella trichopoda*	9	1	2	1	2	1	2		
*Spirodela polyrhiza*	18	1	3	1	2	2	3	6	
*Zostera marina*	19	1	4	0	2	1	1	10	
*Oryza sativa*	24	1	2	1	3	2	5	10	
*Aponogeton distachyos*	21	1	3	1	3	2	3	8	
*Hordeum vulgare*	26	0	3	1	6	1	6	9	
*Chenopodium quinoa*	16	2	4	2	2	4	2	0	
*Glycine max*	37	2	7	1	4	3	4	16	
*Brassica rapa*	21	2	3	1	4	2	3	6	
*Arabidopsis lyrata*	18	1	3	1	2	2	3	6	
*Arabidopsis thaliana*	18	1	3	1	2	2	3	6	

**Table 2:** HSP70s in quinoa and their counterparts in Arabidopsis.

HSP70s in *C.quinoa*		HSP70s in *A.thaliana*		
Quinoa proteome ID	GeneBank ID	TAIR ID	GeneBank ID	similar expression pattern
AUR62024598	XP_021768679.1	AT4G16660	NP_567510.1	yes
AUR62011754	XP_021769992.1	AT4G16660	NP_567510.1	-
AUR62012697	XP_021719698.1	AT1G11660	NP_172631.2	no
AUR62021966	XP_021766890.1	AT1G11660	NP_172631.2	no
AUR62011629	XP_021731972.1	AT1G79930	NP_178111.1	yes
AUR62016444	XP_021736828.1	AT1G79930	NP_178111.1	-
AUR62041322	XP_021729859.1	AT2G32120	NP_180771.1	yes
AUR62024018	XP_021732896.1	AT2G32120	NP_180771.1	no
AUR62032879	XP_021747611.1	AT5G09590	NP_196521.1	no
AUR62005887	XP_021747614.1	AT5G09590	NP_196521.1	yes
AUR62040617	XP_021743068.1	AT5G49910	NP_199802.1	yes
AUR62040144	XP_021719138.1	AT5G49910	NP_199802.1	yes
AUR62023527	XP_021760397.1	AT5G49910	NP_199802.1	yes
AUR62004581	XP_021748808.1	AT5G49910	NP_199802.1	yes
AUR62005192	XP_021742552.1	AT5G42020	NP_851119.1	yes
AUR62000777	XP_021772891.1	AT5G42020	NP_851119.1	no

## Supplementary Materials

The following are available online at www.mdpi.com/2073-4425/9/2/35/s1, Table S1: Primer list for qPCR; File S1: Multiple sequence alignments of CqHSP70 proteins; File S2: Raw Newick file for Figure 2. Figure S1: Electrophoresis result with extracted plant total RNA.
